# Interventions in randomised controlled trials in surgery: issues to consider during trial design

**DOI:** 10.1186/s13063-015-0918-4

**Published:** 2015-09-04

**Authors:** Natalie S. Blencowe, Julia M. Brown, Jonathan A. Cook, Chris Metcalfe, Dion G. Morton, Jon Nicholl, Linda D. Sharples, Shaun Treweek, Jane M. Blazeby, Members of the MRC Hub for Trials Methodology Research Network Workshop

**Affiliations:** Centre for Surgical Research, School of Social and Community Medicine, University of Bristol, Canynge Hall, 39 Whatley Road, Clifton, Bristol, BS8 2PS UK; Division of Surgery, Head & Neck, University Hospitals Bristol NHS Foundation Trust, Bristol, UK; Leeds Institute for Clinical Trials Research, University of Leeds, Clarendon Road, Leeds, UK; Centre for Statistics in Medicine, Nuffield Department of Orthopaedics, Rheumatology and Musculoskeletal Sciences, University of Oxford, Oxford, UK; Academic Department of Surgery, School of Cancer Sciences, Queen Elizabeth Hospital University of Birmingham, Edgbaston, Birmingham, UK; School of Health and Related Research, University of Sheffield, Regent Court, 30 Regent Street, Sheffield, UK; Health Services Research Unit, University of Aberdeen, 3rd Floor, Health Sciences Building, Foresterhill, Aberdeen, UK

**Keywords:** Surgical trials, Trial design, Complex interventions, Methodology, Standardisation, Adherence/fidelity, Expertise

## Abstract

Until recently, insufficient attention has been paid to the fact that surgical interventions are complex. This complexity has several implications, including the way in which surgical interventions are described and delivered in trials. In order for surgeons to adopt trial findings, interventions need to be described in sufficient detail to enable accurate replication; however, it may be permissible to allow some aspects to be delivered according to local practice. Accumulating work in this area has identified the need for general guidance on the design of surgical interventions in trial protocols and reports. Key issues to consider when designing surgical interventions include the identification of each surgical intervention and their components, who will deliver the interventions, and where and how the interventions will be standardised and monitored during the trial. The trial design (pragmatic and explanatory), comparator and stage of innovation may also influence the extent of detail required. Thoughtful consideration of surgical interventions in this way may help with the interpretation of trial results and the adoption of successful interventions into clinical practice.

## Background

There has been an increasing recognition over the past decade that surgical interventions are complex [1–3]. Complex interventions have multiple components, which can act independently or inter-dependently to influence outcomes [3]. The components of surgical interventions may include parts of the operation such as incising, resecting or closing. Surgical interventions are accompanied by concomitant interventions that can occur before, during or after surgery and also have several components - for example, delivering an anaesthetic or providing post-operative pain relief. Contextual factors such as the operating theatre environment, availability of certain equipment and the sheer volume of cases undertaken within a hospital can all influence outcomes and therefore contribute to the complexity. Finally, complex interventions such as surgery can be dependent on the skills of those delivering care (operator expertise), and this may also influence outcomes [4]. Within randomised controlled trials (RCTs) of surgical interventions, it is therefore necessary to consider the surgical and concomitant interventions, contextual factors and operator expertise, and decide to what extent they need to be described and standardised within trial protocols [5].

The MRC (Medical Research Council) guidance for developing and evaluating complex interventions recommends that centres participating in RCTs ‘consistently provide as close to the same intervention as possible’ by ‘standardising the content and delivery of the intervention’. The guidance recommends identifying and piloting the key components of interventions before full evaluation within RCTs [6–8]. Whilst this may be helpful advice, how to operationalise this when designing RCTs in surgery remains unclear. The recent publication of the SPIRIT statement (Standard Protocol Items: Recommendations for Interventional Trials) [9] provides some guidance for describing interventions within trial protocols. It recommends that interventions are ‘described in detail so that they can be replicated in practice’, and states that details about ‘relevant concomitant care and interventions’ and ‘study settings where data will be collected’ be provided. Whether this means that every detail of a surgical operation, anaesthetic and aftercare should be described in the trial protocol is unclear. Providing such detailed descriptions may help to distinguish between the interventions in each trial group, ensuring that they are actually different from one another. For this reason, it may also improve systematic reviews of RCTs of similar surgical interventions. Conversely, however, it is possible that this level of detail is not always necessary or practical. Within large scale pragmatic trials, for example, less information may be required. Detailed descriptions may also fail to reflect the inherent variation in clinical practice and consequently reduce the generalisability of the results. A balance between ‘adequate’ descriptions of interventions and the practicality of delivery is necessary. Some guidance regarding how to go about this process when designing surgical interventions is needed.

The aim of this paper is to describe the key questions to consider during the design of an RCT involving surgical interventions. Issues will be illustrated with examples. This work arose from a 2-day MRC Hub for Trials Methodology Research Network workshop held in June 2013 led by the ConDuCT (Collaboration and Innovation in Difficult Randomised Controlled Trials, Bristol) and Biostatistics (Cambridge) Hubs.

## Review

### Designing surgical interventions within RCTs

Questions to consider during the design of surgical interventions in RCTs are summarised in Table 1, with an accompanying glossary of terms in Table 2. Each item in Table 1 is illustrated below using the example of interventions for appendicitis. This example was chosen because appendicectomy is a common operation with several possible technical variations, and there is an established non-surgical alternative treatment (treatment with antibiotics). The questions address i) surgical interventions (items 1 and 2); ii) concomitant interventions (item 3); iii) how to describe, standardise and monitor surgical and concomitant interventions (items 4, 5 and 6); iv) operator expertise (item 7); and v) context (item 8).

**Table 1:** Questions to ask during the design of surgical interventions in randomised controlled trials (RCTs)

	Description
1	Does the RCT involve a surgical intervention?
2	What is/are the surgical intervention(s) under evaluation?
3	What is/are the concomitant intervention(s) accompanying surgery?
4	What will influence standardisation of the interventions?
	a. What is the overall study design?
	b. What type of comparator is in the RCT?
	c. In what stage of development is/are the surgical intervention(s)?
5	How will the intervention(s) be standardised in the RCT?
6	How will delivery of the intervention(s) be monitored in the RCT (fidelity)?
7	Who will deliver the intervention(s) (operator expertise)?
8	Where will the intervention(s) be delivered (context)?

**Table 2:** Interventions in surgical randomised controlled trials (RCTs): suggested terminology and definitions

Term	Definition
Complex intervention	An intervention with multiple components that act inter-dependently or independently to influence outcomes [6].
Surgical intervention	An intervention that cuts or physically alters a patient’s tissues (whether using a scalpel, stapler, laser or another instrument or device) and involves the use of a sterile environment, anaesthesia, antiseptic conditions and suturing or stapling [10].
Concomitant intervention (or co-intervention)	Interventions that naturally accompany or are associated with the intervention itself [22]. Concomitant interventions can occur before, during or after the main intervention.
Context	The distinctive features of an intervention’s setting, participants and delivery [23].
Expertise	The ability to integrate technical and non-technical skills to complete challenging tasks.
Fidelity	How far those responsible for delivering an intervention actually adhere to the intervention as it is outlined by its designers [24]. Fidelity is also referred to as compliance or adherence.
Pragmatic trial	A trial which is designed to answer the question ‘How well does the intervention work in comparison to the control when delivered under *usual* conditions?’ [25] (that is, effectiveness focused, usually aiming to influence health policy)
Explanatory trial	A trial that is designed to answer the question ‘How well does the intervention work in comparison to the control when delivered under *ideal* conditions?’ [25] (that is, efficacy focused, usually aiming to investigate the causal relationship between an intervention and physiological processes)

### Does the RCT involve at least one surgical intervention?

Initially, it is important to decide if the study in question is a surgical RCT. In this paper, a RCT in surgery is defined as evaluating a surgical intervention in at least one of the trial groups. A surgical intervention is defined as one that involves physically changing body tissues and organs through manual operation such as cutting, suturing, abrading or the use of lasers [10]. Surgical interventions may be the main intervention under investigation or be a concomitant intervention. An example of the former might be the evaluation of open versus laparoscopic surgery for the treatment of appendicitis. Conversely, a trial comparing two different types of post-operative pain relief following surgery for appendicitis would represent an example of surgery as a concomitant intervention (because the main comparison is between the methods of pain relief rather than surgery). This paper will focus on issues to consider in the design of surgical interventions in RCTs in which they are the main intervention under evaluation. Work investigating how concomitant surgical interventions might be described in trial protocols will be addressed separately.

### What are the surgical interventions under evaluation?

A recent systematic review examining reporting standards for surgical interventions found that 30 % of trials reported only the name of the intervention under investigation and gave no further written detail [11]. Providing just the name of a surgical intervention, such as ‘appendicectomy’, leaves this open to different interpretations by different surgeons. It is therefore recommended that specific details of the intervention be clarified and agreed at the beginning of a trial, and described in more detail in trial publications. One way of achieving this is for the trial team to identify systematically all of the constituent components and steps of the surgical intervention during the trial design or pilot work. Examples of potential components and steps involved in performing an appendicectomy are described in Table 3. This identification process should also include any planned further operations; for example, elective two-staged liver resection involves two operations with an intervening interval of between 2 and 6 weeks. It is necessary to clearly state that there is a planned second operation, as well as the anticipated interval between the first and second operations.

**Table 3:** Examples of the steps within a surgical intervention: appendicectomy

Components	Definition	Description	Steps within each component
			Two further 0.5-cm incisions in the left iliac fossa and suprapubic regions.
		Separation and incision of the fat, fascia, muscular layers and peritoneum.	Identification of the base of the umbilicus.
			Incision of fascia using a knife and peritoneum using scissors.
		Creation of a pneumoperitoneum	Insertion of the laparoscopic ports (through fascia and peritoneum).
			Insufflation of gas into the abdomen.
Dissection of the appendix	The process of exposing an organ, tissue or structure	Dissection of tissues surrounding the appendix.	Use of a laparoscopic hook and diathermy to dissect the appendix from surrounding tissues.
		Isolation and ligation of the blood supply.	Use of hook diathermy to divide the mesoappendix and coagulate the artery.
Resection of the appendix	Removal of all or part of an organ, tissue, or structure	Securing the appendix base with staples/sutures.	Application of sutures to the base of the appendix.
		Division of the base of the appendix.	Cutting of the appendix from its base with laparoscopic scissors.
		Removal of the appendix from theabdomen.	Placement of a bag into the abdomen within which to retrieve the appendix through a laparoscopic port.
Closure of the abdomen	Closure of the abdominal layers	Closure of the peritoneum, fascia and skin.	Closure of the fascia at the umbilical port site using a box stitch. Closure of the skin at all three port sites, using sutures or steri-strips.

Systematic documentation of all intervention components, from skin incision through to wound closure, would prevent potentially important aspects of the intervention from being overlooked. Additionally, the *a priori* identification of intervention details will help the trial team to clarify which components and/or steps are considered important to the research question, or the quality assurance of surgery. It will be essential to document and justify the extent of standardisation and monitoring required for such components/steps during the trial (see questions 4 and 5).

### What are the concomitant interventions accompanying the surgical intervention?

Similar to surgical interventions themselves, concomitant interventions often comprise multiple components and these may require consideration during trial design. All of the potential concomitant interventions associated with the surgical intervention may need to be identified and listed during trial team meetings during the design phase (Table 4). Subsequently, the ‘key’ concomitant interventions (and their components) can be detailed within the trial protocol. This may include discussions about which of the concomitant interventions need to be standardised across all trial groups. Factors influencing the level of detail required are discussed below.

**Table 4:** Examples of concomitant interventions accompanying a surgical intervention: knee replacement

Pre-, peri- or post-operative	Concomitant interventions	Description
Pre-operative	Magnetic resonance imaging	Pre-operative imaging to plan the exact type of implant required
	Investigations within the pre-operative assessment clinic	Pre-operative blood tests, electrocardiogram, X-rays and other interventions (such as echocardiogram) as required
	Physiotherapy	Muscle strengthening exercises
Peri-operative	Application of pulse oximeter, oxygen mask and blood pressure cuff	Intra-operative monitoring devices
	Application of continuous pump devices and compression stockings	Preventative measures for deep vein thrombosis
	Insertion of a urinary catheter	To prevent urinary retention (potential consequence of spinal anaesthesia)
	Insertion of a peripheral cannula	To allow delivery of intravenous analgesia
	Administration of spinal anaesthesia	Injection of a local anaesthetic into the subarachnoid space, through a fine needle
	Administration of intravenous analgesia	To minimise intra-operative pain
	Application of a knee bandage	To provide pressure and minimise swelling
Post-operative	Administration of subcutaneous clexane	Preventative measures for deep vein thrombosis
	Application of continuous pump devices and compression stockings	Preventative measures for deep vein thrombosis
	Administration of oral analgesia	To minimise post-operative pain
	Physiotherapy	Muscle strengthening exercises and learning to walk with crutches or a frame
	Use of a continuous passive movement machine	Provide passive motion in a specific plane of movement, and protect the healing repair or tissue

### What will influence the amount of standardisation needed for the intervention?

Key questions to consider are illustrated with published examples in Table 5.

**Table 5:** Examples of published randomised controlled trials (RCTs) in surgery requiring different amounts of standardisation of the interventions

Factors contributing to the level of control required	Example of RCTs requiring less standardisation	Example of RCTs requiring more standardisation
Scope of the trial	Comparison of surgery and medical therapy for treatment of gastro-oesophageal reflux disease [26]	Carotid artery stenting compared with endarterectomy in patients with carotid stenosis [27]
Pragmatic versus explanatory approach to design	Pragmatic: The trial aimed to establish the effectiveness of the interventions in practice	Explanatory: The trial aimed to establish the safety and efficacy of the ‘new’ intervention (stenting) under ideal conditions
Stage of innovation of the intervention	Open versus minimally invasive surgery for colorectal cancer [28]	Endovascular versus open repair of abdominal aortic aneurysms [29]
Established versus new innovation	Established: Open colorectal surgery is routinely undertaken by many operators in clinical practice and the main steps are already known	Early: Endovascular aneurysm repair is a relatively new technique and the main steps are less well known
Complexity of the intervention	Effect of an implantable gentamicin-collagen sponge on sternal wound infections following cardiac surgery [30]	Extracranial-intracranial bypass surgery for stroke prevention in hemodynamic cerebral ischemia [31]
Few components and low technical difficulty versus multiple components or requiring highly complex skills	Low complexity: Intervention involves placing an antibiotic impregnated sponge beneath patients’ wounds prior to closure	High complexity: Intervention is complex involving multiple components, high levels of technical expertise and multiple concomitant interventions
Nature of interventions being compared	Amoxicillin plus clavulanic acid versus appendicectomy for treatment of acute uncomplicated appendicitis [32]	Laparoscopic gastric bypass versus laparoscopic duodenal switch for super obesity [33]
Surgical and non-surgical intervention, or different surgical interventions	Surgery versus non-surgery: Distinction between trial groups is straightforward because of the very different nature of interventions	Surgery versus surgery: Distinction between the two groups is less straightforward because both interventions are surgical

#### What is the overall trial design?

The overall design and purpose of the trial will influence the extent to which interventions are standardised. Explanatory trials (defined in Table 2), which determine the efficacy of interventions, are likely to require detailed intervention descriptions and strict standardisation of delivery. Because explanatory trials often involve just a few centres or surgeons, tend to have a less varied patient cohort, and may be conducted by the surgeons responsible for developing the novel procedure, this may be possible to achieve. Conversely, pragmatic designs (defined in Table 2) determine whether interventions are effective in a more general setting, and therefore usually involve large numbers of surgeons and centres. Under such circumstances, ensuring tight control over intervention delivery is likely to be difficult and may be undesirable given the pragmatic design. Although a less detailed description of the intervention may be required in the trial protocol, those components that are considered to be ‘key’, or those that are crucial in distinguishing the interventions between trial groups, should be reported as a minimum.

#### What type of comparator is in the study?

It is important to establish how the surgical intervention under investigation is different to treatment within the other trial groups. For example, in a trial comparing a surgical and non-surgical intervention (surgery versus antibiotics for appendicitis), a detailed description of the operation may not be required because the key difference between the interventions are immediately apparent. In a trial comparing two perations for appendicitis (for example, laparoscopic and single-port appendicectomy), the interventions are more similar to each other. It may therefore be important to clarify the expected differences in intervention delivery between the two groups (for example, the number and size of laparoscopic ports), to describe these in the protocol and monitor their delivery during the trial.

#### What stage of development is the intervention?

The stage of development of an intervention may influence the amount of detail required in the trial protocol. The IDEAL (Idea-Development-Exploration-Assessment-Long term study) guidance provides a framework for the staged development and evaluation of novel surgical interventions [1–3]. In stage 2b, pilot work is recommended prior to rolling out an effectiveness trial because the intervention may be still evolving. In this type of pilot study it is likely that extensive detail regarding the intervention needs to be documented and strict standardisation of the intervention mandated, along with explicit reporting of any changes implemented and the rationale behind them. In IDEAL Stage 3 RCTs, the extent to which interventions need to be described and standardised may depend more upon the overall trial design (pragmatic versus explanatory approaches - see above).

### How will the intervention(s) be standardised in the RCT?

Standardisation of surgical interventions relates to the extent to which trial protocols specify exactly how they should be delivered. Several factors may influence the amount of standardisation that may be required, such as the overall trial design and research objective, and these are considered in question 4. The process of deciding how much standardisation is necessary starts during trial design, with the trial team identifying all of the constituent components and steps of each intervention (questions 2 and 3). Once the components and steps have been listed, details about exactly how they should be performed can be established. For example, trialists can decide whether each of the components and steps are mandatory, prohibited or optional [12, 13]. Subsequently, they can consider whether the components and steps should be performed in a specific way, or flexibly (that is, according to individual surgeon preferences). An example of a trial in which standardisation of the surgical interventions was unclear, subsequently leading to a published debate, is provided in Table 6.

**Table 6:** An example of a surgical randomised controlled trial (RCT) in which the surgical interventions were questioned following publication of the trial

Trial features	
Journal, year of publication	Lancet, 2009
Aim of RCT	To compare survival between standard or minimally invasive surgery in patients with colorectal cancer [28].
Main findings	The survival rate following minimally invasive surgery was non-inferior to standard surgery
Verbatim description of the surgical intervention	The following description was provided for both open and minimally invasive surgery: ‘Resection involved the division of blood vessels and bowel’. Conversion to open surgery was defined as a ‘vertical incision’. No other description regarding the incisions was provided.
Published correspondence	‘What about right-sided cancers? It is well known among laparoscopic surgeons that ‘laparoscopic’ right colectomy can in fact mean three different kinds of procedures: a true or totally laparoscopic right colectomy (with both dissection and anastomosis done intra-corporeally), laparoscopically assisted colectomy (with only the vascular ligation done intra-corporeally), or so-called laparoscopic colectomy (with only the colonic dissection done laparoscopically). The authors of the CLASICC trial stated only that resection involved ‘(where possible) the division of blood vessels and bowel’. Conversion was defined as a ‘vertical incision’, but what about (for example) a transverse incision of almost 7 cm in a thin patient allowing both division of vessels and bowel after a simple laparoscopic colonic mobilisation? Is that procedure laparoscopic or open? Since the actual procedure is not specified, the external validity of trials regarding the laparoscopic approach for right-sided colon cancers should be questioned [34].’

It may be very difficult to standardise completely the ‘form’ of complex interventions such as surgery - that is, all of the components. One potential compromise would be to standardise an intervention’s key ‘functions’ rather than specifying the specific ‘form’ it needs to take. Standardisation of surgical interventions in trial protocols may, therefore, range from completely flexible delivery of the entire intervention to rigid standardisation of every step. In appendicectomy, for example, it may simply be stated that the appendix be removed (that is, standardisation of ‘function’ only), meaning this can be performed with robotic or minimal access techniques, or using an open incision. Even if complete flexibility is allowed, this should be stated explicitly to avoid doubt. The other extreme - where an intervention is rigidly standardised in a trial - requires extensive details to be provided within the trial protocol. This would include information about each of the mandatory, optional and prohibited components and steps of the intervention (that is, standardisation of ‘function’ and ‘form’) and delivery of these would be monitored strictly during the trial itself.

### Will intervention delivery be monitored during the trial? If so, how?

During a trial, it may be necessary to assess whether the intervention was delivered as intended (fidelity). Fidelity has been defined as ‘how far those responsible for delivering an intervention actually adhere to the intervention as it is outlined by its designers’ [14]. The degree to which intervention fidelity needs to be monitored within RCTs will largely depend upon the extent to which the trial protocol prescribes standardisation. For example, if it is decided that certain components or steps of an operation are essential, monitoring of these components to see if they are delivered as planned will be important. In addition, it may be necessary to monitor whether prohibited steps were performed in error during the trial. Assessing whether key components of an intervention are actually performed according to protocol can be challenging and may require video or photographic evidence, direct observation or surgeon-reported descriptions of what was done [11]. If fidelity is found to be lacking within a trial taking an explanatory approach, active steps might be taken to improve this including additional training or withdrawal of surgeons. Conversely, in trials with a more flexible protocol, monitoring of the delivery of interventions may not be required, particularly if the trial protocol clearly states that these steps can be carried out according to surgeons’ preferred techniques. One limitation of this pragmatic approach would be the potential loss of documentation of fidelity, which can be helpful in summarising the range of techniques used and in interpretation of trial results.

### Who will deliver the interventions?

Surgical interventions are not usually delivered by a single surgeon working in isolation. Instead, they are typically supported by one or more surgeon colleagues and a surgical team (for example, nurses and anaesthetists). The skills of all these team members may influence treatment outcomes, and it may be necessary to account for them in RCTs evaluating surgical interventions, both at the design and analysis stage [15]. Some surgical interventions may be more dependent on particular expertise than others (see question 4), and a discussion of these issues during trial design is recommended. If the delivery of a surgical intervention is considered to be heavily dependent on expertise, or requires a step-change in technical skill, it may be necessary to set pre-defined entry criteria for surgeons such as a minimum annual caseload, attendance of training courses, number of years in training, or outcome data thresholds [11]. Alternatively, it may be possible to account for expertise within trial design using expertise-based RCTs [16] or the detection and modelling of learning curves [17]. In other circumstances, for example more pragmatic trial designs, formal minimal criteria for surgeons’ expertise beyond what is required in routine practice may be of limited value. This is contentious, however, as some consider that trials should never involve ‘novice’ surgeons who are still learning techniques, irrespective of the overall design, because the results may be interpreted as less convincing.

### Where will the interventions be delivered?

Context can be defined as the distinctive features of an intervention’s setting, participants and delivery [18]. In surgical RCTs, this may include the type of hospital and department (for example, tertiary centre versus district general hospital) as well as features of the surgical teams (for example, specialists versus generalists). Whilst context may be more relevant in some RCTs than others (see question 4), it is recommended that all trials consider and describe whether there are any elements of context that were important for the delivery of the trial or that may have had an impact on the effect of the intervention. In some circumstances, it may be necessary to set pre-defined criteria regarding context, such as centre volume or constituent members of the multi-disciplinary team responsible for delivering the surgical interventions.

## Discussion

This paper identifies key questions relevant to the design and reporting of surgical interventions in RCTs in surgery. The questions cover issues relating to intervention description, standardisation and monitoring, expertise of surgical teams, and the context of intervention delivery. Consideration of these issues is recommended so that appropriate levels of detail about interventions, and methods for monitoring their delivery, can be documented in the trial protocol and can be subsequently assessed during the trial itself. This may go some way to reduce the criticisms currently levelled at the heterogeneous delivery of interventions within RCTs in surgery.

Since the workshop in which these issues were discussed took place, the TIDieR (Template for Intervention Description and Replication) checklist has been published [19]. TIDieR provides guidance for describing interventions within RCTs, recommending that information other than just the name of the intervention be reported. The authors state that ‘the overarching purpose of the TIDieR checklist is to prompt authors to describe interventions in sufficient detail to allow their replication’. Items within TIDieR include consideration of the processes involved in the intervention, the mode of delivery and any context and expertise requirements. Although these items are helpful in surgical settings, TIDieR is not specific to a particular intervention type. This means that some of the checklist items may be less relevant to surgery, for example, intervention duration, dose and the number of times delivered. Additionally, there may be important omissions for describing complex interventions. For example, TIDieR does not include concomitant interventions that may be particularly relevant for interventions such as surgery. Although the guidance states that interventions should be described in sufficient detail to allow replication, it does not discuss potential circumstances that may remove the need for such in-depth information, such as study design (for example, pragmatic versus explanatory approach) or the stage of innovation of the intervention (for example, ‘established versus ‘new’ procedures).

This paper recommends that trialists list all the components and steps of surgical interventions in a thorough and logical way, and then identify which components need to be standardised. It does not, however, explore how the key components might be identified if they are not obvious after all of the intervention components and steps have been listed. Whilst this can be informed by discussion and meetings, it is an area that needs further work. There are several potential methods for determining the key components of surgical interventions. These include surveys of literature and current practice, consensus methods to reach agreement between stakeholders, the use of process evaluations or a combination of all three. To date, however, there has been limited reporting of these methods in surgical settings and further research is required to ascertain their applicability and relevance [20, 21].

## Conclusions

Randomised controlled trials in surgery can be difficult to design and conduct, and one of the reasons for this is that surgical interventions are complex. This complexity needs to be accounted for during trial design by considering how surgical interventions might be described, standardised and monitored. This paper provides practical guidance for surgeons and trialists to use when designing interventions in surgical RCTs to aid them in this process, so this information can be included *a priori* within trial protocols. This may help with the interpretation of trial results and the adoption of successful interventions into clinical practice. It is now necessary to test this guidance during the design phases of new surgical trials to assess its usability, usefulness and acceptability.

## Abbreviations

MRC: Medical Research Council
IDEAL: Idea-Development-Exploration-Assessment-Long term study
RCT: randomised controlled trial
SPIRIT: Standard Protocol Items: Recommendations for Interventional Trials
TIDieR: Template for Intervention Description and Replication

## References

[1] Barkun JS, Aronson JK, Feldman LS, Maddern GJ, Strasberg SM, Altman DG (2009). Evaluation and stages of surgical innovations. Lancet..

[2] Ergina PL, Cook JA, Blazeby JM, Boutron I, Clavien PA, Reeves BC (2009). Challenges in evaluating surgical innovation. Lancet..

[3] McCulloch P, Altman DG, Campbell WB, Flum DR, Glasziou P, Marshall JC (2009). No surgical innovation without evaluation: the IDEAL recommendations. Lancet..

[4] Walwyn R, Roberts C (2010). Therapist variation within randomised trials of psychotherapy: implications for precision, internal and external validity. Stat Methods Med Res..

[5] Wells M, Williams B, Treweek S, Coyle J, Taylor J (2012). Intervention description is not enough: evidence from an in-depth multiple case study on the untold role and impact of context in randomised controlled trials of seven complex interventions. Trials..

[6] Medical Research Council (2008). Developing and evaluating complex interventions: new guidance.

[7] Craig P, Dieppe P, Macintyre S, Michie S, Nazareth I, Petticrew M (2008). Developing and evaluating complex interventions: the new Medical Research Council guidance. BMJ..

[8] Anderson R (2008). New MRC, guidance on evaluating complex interventions. BMJ..

[9] Chan AW, Tetzlaff JM, Gotzsche PC, Altman DG, Mann H, Berlin JA (2013). SPIRIT 2013 explanation and elaboration: guidance for protocols of clinical trials. BMJ..

[10] Cook JA (2009). The challenges faced in the design, conduct and analysis of surgical randomised controlled trials. Trials..

[11] Blencowe NS, Boddy AP, Harris A, Hanna T, Whiting P, Cook JA (2015). A systematic review of intervention design and delivery within pragmatic and explanatory surgical RCTs.

[12] Blencowe N, Mills N, Whiting P, Blazeby J (2013). Providing adequate and practical descriptions in surgical trials. BMJ..

[13] Avery KN, Barham CP, Berrisford R, Blazeby JM, Blencowe NS, Donovan J (2013). Understanding surgical interventions in RCTs: the need for better methodology. Lancet..

[14] Carroll SL, McGillion M, Stacey D, Healey JS, Browne G, Arthur HM (2013). Development and feasibility testing of decision support for patients who are candidates for a prophylactic implantable defibrillator: a study protocol for a pilot randomized controlled trial. Trials..

[15] Papachristofi O, Mackay JH, Powell SJ, Nashef SA, Sharples L (2014). Impact of the anesthesiologist and surgeon on cardiac surgical outcomes. J Cardiothorac Vasc Anesth..

[16] Devereaux PJ, Bhandari M, Clarke M, Montori VM, Cook DJ, Yusuf S (2005). Need for expertise based randomised controlled trials. BMJ..

[17] Harrysson IJ, Cook J, Sirimanna P, Feldman LS, Darzi A, Aggarwal R (2014). Systematic review of learning curves for minimally invasive abdominal surgery: a review of the methodology of data collection, depiction of outcomes, and statistical analysis. Ann Surg..

[18] Treweek S, Zwarenstein M (2009). Making trials matter: pragmatic and explanatory trials and the problem of applicability. Trials..

[19] Hoffmann TC, Glasziou PP, Boutron I, Milne R, Perera R, Moher D (2014). Better reporting of interventions: template for intervention description and replication (TIDieR) checklist and guide. BMJ..

[20] Randell R, Greenhalgh J, Hindmarsh J, Dowding D, Jayne D, Pearman A (2014). Integration of robotic surgery into routine practice and impacts on communication, collaboration, and decision making: a realist process evaluation protocol. Implementation science : IS.

[21] Blencowe N, Mills N, Donovan J, Blazeby J (2013). Understanding the complexity of surgical interventions in rcts: the role of process evaluation in the operating theatre. Trials.

[22] Avery KN, Metcalfe C, Berrisford R, Barham CP, Donovan JL, Elliott J (2014). The feasibility of a randomised controlled trial of minimally invasive and open surgery for oesophageal cancer - the ROMIO (randomised oesophagectomy: minimally invasive or open) study: study protocol for a randomised controlled trial. Trials..

[23] Rychetnik L, Frommer M, Hawe P, Shiell A (2002). Criteria for evaluating evidence on public health interventions. J Epidemiol Community Health..

[24] Carroll C, Patterson M, Wood S, Booth A, Rick J, Balain S (2007). A conceptual framework for implementation fidelity. Implementation science: IS..

[25] Thorpe KE, Zwarenstein M, Oxman AD, Treweek S, Furberg CD, Altman DG (2009). A pragmatic-explanatory continuum indicator summary (PRECIS): a tool to help trial designers. J Clin Epidemiol..

[26] Grant AM, Cotton SC, Boachie C, Ramsay CR, Krukowski ZH, Heading RC (2013). Minimal access surgery compared with medical management for gastro-oesophageal reflux disease: five year follow-up of a randomised controlled trial (REFLUX). BMJ..

[27] Ferguson GG, Eliasziw M, Barr HWK, Clagett GP, Barnes RW, Wallace MC (1999). The North American symptomatic carotid endarterectomy trial: surgical results in 1415 patients. Stroke..

[28] Guillou PJ, Quirke P, Thorpe H, Walker J, Jayne DG, Smith AMH (2005). Short-term endpoints of conventional versus laparoscopic-assisted surgery in patients with colorectal cancer (MRC CLASICC trial): multicentre, randomised controlled trial. Lancet..

[29] Investigators UKET, Greenhalgh RM, Brown LC, Powell JT, Thompson SG, Epstein D (2010). Endovascular versus open repair of abdominal aortic aneurysm. N Engl J Med..

[30] Bennett-Guerrero E, Ferguson T, Lin M, Garg J, Mark DB, Scavo VA (2010). Effect of an implantable gentamicin-collagen sponge on sternal wound infections following cardiac surgery: a randomized trial. JAMA..

[31] Powers WJ, Clarke WR, Grubb RL, Videen TO, Adams HP, Derdeyn CP (2011). Extracranial-intracranial bypass surgery for stroke prevention in hemodynamic cerebral ischemia: The carotid occlusion surgery study randomized trial. JAMA..

[32] Vons C, Barry C, Maitre S, Pautrat K, Leconte M, Costaglioli B (2011). Amoxicillin plus clavulanic acid versus appendicectomy for treatment of acute uncomplicated appendicitis: an open-label, non-inferiority, randomised controlled trial. Lancet..

[33] Sovik TT, Taha O, Aasheim ET, Engstrom M, Kristinsson J, Bjorkman S (2010). Randomized clinical trial of laparoscopic gastric bypass versus laparoscopic duodenal switch for superobesity. Br J Surg..

[34] Slim K (2005). MRC CLASICC trial. The Lancet.

